# Continuous versus discontinuous suture in perineal injuries produced during delivery in primiparous women: a randomized controlled trial

**DOI:** 10.1186/s12884-019-2655-2

**Published:** 2019-12-16

**Authors:** Juan Miguel Martínez-Galiano, Beatriz Arredondo-López, Leticia Molina-Garcia, Ana Maria Cámara-Jurado, Eva Cocera-Ruiz, Miguel Rodríguez-Delgado

**Affiliations:** 1 Department of Nursing, University of Jaen, Jaen, Spain; 2 Consortium for Biomedical Research in Epidemiology and Public Health (CIBERESP), Madrid, Spain; 3 Andalusian Health Service, Jaen, Spain; 4 Department of Health Sciences, University of Jaen, Jaen, Spain

**Keywords:** Suture, Perineal lesion, Episiotomy, Incontinence, Pain, Sexual relations

## Abstract

**Background:**

The technique used in the repair of a perineal injury resulting from childbirth could avoid discomfort and morbidity during the postpartum period. Recent studies show inconsistent results and support the need for new research with the inclusion of new health parameters not yet studied. Therefore, this study aims to evaluate if the suture technique (continuous or interrupted) has an effect on pain and other postpartum problems, incidence of incontinence (urinary and/or fecal), and the restart of sexual relations.

**Methods:**

A single-blind randomized clinical trial was conducted in five hospitals in south-east Spain. The participants were primiparous women who had experienced a perineal injury during delivery (second-degree tear or episiotomy). Data was collected on sociodemographic variables, variables associated with pregnancy, labor and delivery, and the postpartum period, and outcomes during the 3 months after delivery: pain, incontinence, and restart of sexual relations. Odds ratios (OR) were calculated by binary logistic regression to assess the influence of the suture type on binary outcomes and t-test used for comparing continuous outcomes. Multivariate analyses (using logistic regression -adjusted (aOR)- and analysis of covariance) were carried out to adjust for unbalanced variables after randomization.

**Results:**

A total of 70 women were included in the intervention group (continuous suture) and 64 in the reference group (interrupted sutures). A negative association was observed (aOR = 0.39; 95% CI = 0.18–0.86) between a continuous suture and the need for analgesia at 24 h postpartum. Pain experienced by the women at 24 h postpartum was assessed as 4.4 ± 0.3 compared with a score of 3.4 ± 0.3 in the group with continuous sutures (*p* = 0.011). At 15 days postpartum, women in the intervention group experienced less pain (aOR = 0.38; 95% CI = 0.18–0.80) (*p* = 0.019). Urinary sphincter incontinence was also evaluated at 15 days, with 4.3% (*n* = 3) of the women in the intervention group presenting with urinary incontinence compared with 18.8% (*n* = 12) in the control group (aOR = 0.11; 95% CI = 0.03–0.47) (*P* = 0.003).

**Conclusions:**

The women who had a continuous suture repair showed lower levels of pain from delivery to 3 months after delivery and had a lower incidence of urinary incontinence at 15 days postpartum.

**Trial registration:**

ClinicalTrials.gov NCT03825211 posted January 31, 2019 (retrospectively registered).

## Background

During childbirth perineal trauma may occur, either spontaneously or after episiotomy [1, 2]. In Spain the actual rate of episiotomies is unknown: According to the available data, it varies between 33 and 73% of all vaginal deliveries [3]. Perineal trauma rates vary considerably according to individual practices and the policies of each hospital. In 2010, a European study reported that the perineal tear rate oscillated between 0.1% in Romania and 4.9% in Iceland, and the episiotomy rate varied between 3.7% in Denmark and 75% in Cyprus [4]. These injuries result in both short- and long-term morbidity, with possible effects on the pelvic floor [5], can cause urinary or anal incontinence, perineal pain, and may also affect women’s sexual function [2, 4–6].

In addition, the type of suture technique used in the perineal lesion repair can also cause maternal morbidity [3, 4]. Different suture techniques exist, with the most used technique being the interrupted suture—closing the skin with separate stitches. In contrast, the continuous suture consists of joining all the tissue layers involved in the perineal lesion with the same suture thread in a continuous manner [3, 7–9].

Different studies have associated the suture technique and type of suture with differing complaints and pathologies in women [3, 7, 8, 10]. In 2008, a randomized controlled trial addressing perineal repair was conducted in Spain where a continuous suture repair used in 233 women and a interrupted suture technique in another 222 women [11]. No significant differences in pain were found between these repair techniques. In contrast, in Turkey a 2009 study conducted on 160 women concluded that a continuous suture technique was associated with less pain in the short-term [12]. A 2012 Cochrane review that included 16 studies also concluded that women who had continuous sutures experienced less pain in the short-term and also required less analgesia [13]. In this review, only pain and the requirement for analgesia in the short-term and at 10-days were taken into account, and it recommended carrying out more research to address the outcomes relevant to women; including sexual problems and pelvic floor muscle dysfunction (such as incontinence) in the immediate period as well as in the long-term after delivery [13]. However, a clinical trial conducted in 2017 in India with 200 women, concluded that there was no difference between the two suture techniques in terms of pain on days 2, 10 and 90: This study did not assess other outcomes [14].

The use of one or another technique in the repair of perineal trauma can prevent morbidity and discomfort to women in the postpartum period. Therefore, the aim of this study was to evaluate whether the suture technique (continuous vs interrupted) had an effect on pain, incidence of incontinence (urinary and/or fecal), and the restart of sexual relations.

## Methods

A prospective, controlled, multicenter clinical trial was conducted, with random allocation of women to two groups, between the months of November 2016 and May 2018.

### Population selection

The reference population was women who gave birth in the following hospitals situated in south-east Spain: San Juan de la Cruz in Ubeda (Jaen), San Agustin in Linares (Jaen), Jaen University Hospital Complex, University Hospital Virgen de las Nieves (Granada), and Hospital Torrecardenas in Almeria.

The inclusion criteria were: Age > 18 years, primiparous, singleton and eutocic delivery, second-degree perineal tear or an episiotomy as part of labor, and a newborn weight between 2500 g and 4000 g. The following exclusion criteria were used: language barrier, problems related to the pelvic floor prior to labor (prolapse, incontinence, vulva varices, etc.), dyspareunia or sexual dysfunction, hemorrhoids perceived as uncomfortable or painful, or women who did not wish to participate or did not sign the informed consent.

**Fig. 1:**
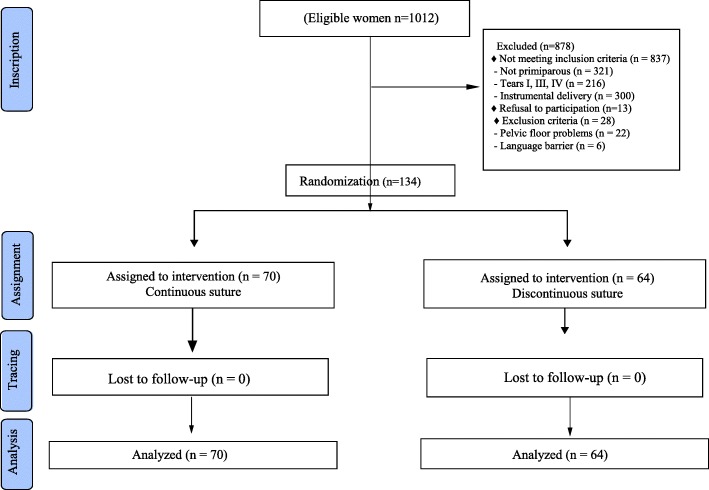
Flow diagram of the participants

To estimate the sample size, the main outcome was taken into account: pain. Sample size estimation was based on the next assumptions: a statistical power of 80%, an α level of 5%, a 1:1 allocation between the intervention group and the reference group, a frequency of pain of 60.4% in women with interrupted sutures [9], and a priori reduction of pain of 50% with continuous sutures. With these assumptions 54 women would be required in each group: Assuming a loss of 20%, the sample size needed was 67 women in each group. Figure 1.

### Intervention

Two types of sutures were used for the perineal injury using two different techniques: Group A received continuous sutures and Group B had interrupted sutures placed. The two techniques and sutures are described in more detail in Additional file 1. The women were allocated to a treatment group using a computer-generated random numbers table. The generated sequence was placed in individual opaque envelopes that were opened when a woman met the inclusion criteria.

The health personnel that placed the sutures had previously taken a training course on continuous suture technique and had at least 1 year of experience in this technique. Additionally, this person had a minimum of 5 years of experience in attending deliveries and, therefore, also in the suturing of perineal injuries. The sutures were placed by ten trained midwives. Around 5% [1] of the midwives in Torrecardenas, 10% [2] in the Hospital Complex of Jaen, 12% [3] in Granada, and 20% [2] of the midwives in Ubeda and Linares participated.

### Data collection

To evaluate the perception of pain experienced by women during labor the verbal rating scale (VRS) was used [15].

Data was collected on sociodemographic variables, type of perineal injury (tear of second-degree or episiotomy), type of suture used, time spent placing suture, number of suture packets used, complications, severity of pain, type of pain, need for analgesia, wound condition, care performed, urinary and/or fecal incontinence, start of sexual relations, and start of physical activity. Data was also collected on the start of labor (spontaneous or induced) and type of delivery, medication during dilation, type of analgesia used, gestational week, duration of the first stage of labor, second stage labor and delivery, as well as data regarding the newborn.

Information was gathered by midwives who interviewed the women in the delivery room, providing them with an informed consent form. The rest of the data were obtained from the clinical history, maternal record book and via follow-up phone calls. The women were blinded to the suture technique used.

### Follow-up

The women were followed-up as follows:

Day of childbirth: in the postpartum period, the midwife collected data on the type of delivery, need for epidural analgesia, duration of dilation, stage two of labor, delivery, type of perineal trauma, time used to place suture, number of suture packets, wound complications, newborn weight and the Apgar score at 1 min and at 5 min. After the suture was placed, they assessed pain on the pain scale and assessed the wound.

Follow-up after delivery was done at 2 h, 24 h, 15 days and 3 months. Pain, need for analgesia, wound condition, sphincter incontinence, and the start of sexual relations were assessed (15 days and 3 months).

### Data analysis

First of all, randomized groups were compared regarding variables which could influence the outcomes: statistically significant imbalances were found for marital status and attendance to a health education course during pregnancy. Associations between binary outcomes (e.g., pain - yes/no, incontinence - yes/no) was ascertained by computing odds ratios (OR) and their 95% confidence intervals (CI); the variables imbalanced after randomization were adjusted for using a non-conditional logistic regression analysis. Normality was checked in continuous variables (e.g., pain assessed by verbal rating scale, time to restart sexual intercourse). In the comparison of continuous outcomes between the two groups the t-test was applied in univariate analyses, and the analysis of covariance to adjust for the two imbalanced baseline variables.

### Ethics approval

The study was carried out following the precepts included in the Belmont report and the Declaration of Helsinki (updated at the Brazil Assembly in 2013) for biomedical research.

Both the design and the development of the work conform to the standards of good clinical practice (CPMP / ICH / 135/95, July 2002 revision of the European Medicines Agency -EMeA).

Ethical approval was obtained from the Ethics Committees of the hospitals participating in the study: Comité de Ética de la Investigación del Hospital Universitario Virgen de las Nieves de Granada (Ethics Committee for Research of the University Hospital Virgen de las Nieves in Granada), Comité de Ética de la Investigación del Hospital Torrecardenas (Ethics Committee for Research of the Torrecardenas Hospital in Almeria), Comité de Ética de la Investigación del Complejo Hospitalario Universitario de Jaén; (Ethics Committee for Research of the University Hospital of Jaen) Comité de Ética de la Investigación del Hospital de Úbeda (Ethics Committee for Research of the Ubeda Hospital) and Comité de Ética de la Investigación del Hospital de Linares (Ethics Committee for Research of the Linares Hospital).

Written informed consent was obtained from all the women before recruitment into the study. Informed consent was obtained from the women participating in the study and we also followed the protocols established by the respective health centres for accessing data from medical records to carry out this type of research with the purpose of publication/disclosure to the scientific community.

(ClinicalTrials.gov Identifier: NCT03825211, restrospectively registered)

## Results

A final total of 70 women were included in the intervention group—who received continuous sutures—and 64 were in included in the reference group.

In Table 1 the two groups are compared according to different sociodemographic, pregnancy, and labor/delivery variables. No significant differences were found between groups for age, education level, body mass index, presence of prior illnesses, or physical exercise during pregnancy. Significant differences were only observed in two variables: civil status and attendance at the education program during pregnancy on labor and newborn care. In the group with interrupted suture there were more single women and also more women attending the educational program on pregnancy, labor and care of newborn. With respect to civil status, 67.2% (*n* = 47) in the continuous suture group were married, compared with 59.4% (*n* = 38) in the interrupted suture group (*p* = 0.047). In terms of attendance at an education program during pregnancy, 54.3% (*n* = 38) of the continuous suture group attended this, compared with 73.4% (*n* = 47) in the interrupted suture group (*p* = 0.031).

**Table 1:** Description of the study population

Variables	Continuous suture n (70)	Interrupted suture n (64)	*P* value
Age (years), m (SEM)^a^	30.98 ± 0.63	30.76 ± 0.68	0.813
Civil status			0.047
Single, n (%)	7 (10.00)	17 (26.56)	
Married, n (%)	47 (67.14)	38 (59.38)	
Stable relationship, n (%)	15 (21.43)	9 (14.06)	
Divorced, n (%)	1 (1.43)	0 (0.00)	
Education level			0.797
Incomplete primary, n (%)	2 (2.86)	1 (1.56)	
Primary, n (%)	3 (4.29)	3 (4.69)	
Incomplete secondary, n (%)	2 (2.86)	4 (6.25)	
Secondary, n (%)	17 (24.29)	18 (28.13)	
Higher secondary education, n (%)	19 (27.14)	12 (18.75)	
University, n (%)	27 (38.57)	26 (40.63)	
BMI^b^, m (SEM)	28.19 ± 0.51	27.27 ± 0.46	0.185
Previous illness, Yes, n (%)	6 (8.57)	8 (12.50)	0.575
Exercise during pregnancy, Yes, n (%)	39 (55.71)	34 (53.13)	0.862
Perineal massage pre-labor, Yes, n (%)	15 (21.43)	12 (18.75)	0.830
Attendance during pregnancy at an education course on pregnancy, delivery and newborn care, Yes, n (%)	38 (54.29)	47 (73.44)	0.031
Gestational age at delivery (weeks), m (SEM)	39.57 (0.13)	39.78 (0.14)	0.275
Start of labor			0.473
Spontaneous, n (%)	48 (68.57)	40 (62.50)	
Induced, n (%)	22 (31.43)	24 (37.50)	
Epidural analgesia, Yes, n (%)	56 (80.00)	53 (82.81)	0.825
Duration of the first stage of labor (minutes), m (SEM)	314.80 (22.87)	342.72 (21.10)	0.373
Duration of the second stage of labor (minutes), m (SEM)	85.61 (6.29)	96.64 (8.23)	0.284
Duration of the third stage of labor (minutes), m (SEM)	9.71 (1.06)	9.70 (1.02)	0.994
Type of perineal lesion			0.387
Episiotomy, n (%)	32 (45.71)	35 (54.69)	
2nd degree tear, n (%)	38 (54.29)	29 (45.31)	
Weight of newborn (grams), m (SEM)	3252.6 (41.8)	3185.1 (50.5)	0.301

^a^ m (SEM): mean (standard error of the mean)

^b^ BMI (body mass Index) = Kg/m^2^

With respect to the duration of the suture, the time to place suture in the intervention group had a mean of 14.1 ± 0.9 min, compared with 18.2 ± 1 min in the reference group (*p* = 0.003). The number of suture packets that were needed to perform the continuous suture was on average of 1.1 ± 0.03 packets, compared with 2.3 ± 0.06 used in the interrupted technique (*p* < 0.001).

Table 2 shows the association between the suture technique that was used and the effects measured as binary variables. Of the women who had an interrupted suture 66.7% (*n* = 42) required analgesia at 24 h, compared to 52.9% (*n* = 37) of those who had continuous sutures; with a negative association observed (aOR = 0.39, 95% CI = 0.18–0.86) (*p* = 0.019). Regarding pain in the perineal area at 15 days postpartum, this was experienced by 31.4% (*n* = 22) in the intervention group, compared to 53.1% (*n* = 34) of the women who underwent interrupted sutures (aOR = 0.38, 95% CI = 0.18–0.80) (*p* = 0.011). Urinary sphincter incontinence was also assessed at 15 days, and was experienced by 4.3% (*n* = 3) of the women in the intervention group vs 18.8% (*n* = 12) in the reference group (aOR = 0.11, 95% CI = 0.03–0.47) (*p* = 0.003). The number of women restarting sexual intercourse was small and no difference was found between the two groups at 15 days, but at 3 months the number of women normalizing their sexual relationships was higher in the intervention group (aOR = 4.78, 95% = 2.14–10.64) (p<0.001).

**Table 2:** Type of suture and effects on the woman (binary variables)

Effect variables	Continuous suture	Interrupted suture	OR (95%CI)	*p*-value	ORa^a^ (95% CI)	*p*-value
Perineal pain at 24 h post delivery				0.087		0.048
Yes	62 (88.47)	62 (96.88)	0.25 (0.05–1.22)		0.17 (0.03–0.99)	
No	8 (11.43)	2 (3.13)	1 (reference)		1 (reference)	
Analgesia at 24 h post delivery				0.107		0.019
Yes	37 (52.86)	42 (66.67)	0.56 (0.28–1.13)		0.39 (0.18–0.86)	
No	33 (47.14)	21 (33.33)	1 (reference)		1 (reference)	
Perineal wound dehiscence at 24 h post delivery				0.871		0.776
Yes	6 (8.57)	6 (9.38)	0.91 (0.28–2.97)		1.20 (0.35–4.11)	
No	64 (91.43)	58 (90.63)	1 (reference)		1 (reference)	
Perineal pain at 15 days post delivery				0.012		0.011
Yes	22 (31.43)	34 (53.13)	0.4 (0.20–0.82)		0.38 (0.18–0.80)	
No	48 (68.57)	30 (46.88)	1 (reference)		1 (reference)	
Analgesia at 15 days post delivery				0.849		0.646
Yes	8 (11.43)	8 (12.50)	0.90 (0.32–2.57)		0.78 (0.26–2.30)	
No	62 (88.57)	56 (87.50)	1 (reference)		1 (reference)	
Perineal wound dehiscence at 15 h post delivery				0.518		0.445
Yes	1 (1.43)	2 (3.31)	0.45 (0.04–5.08)		0.38 (0.03–4.57)	
No	69 (98.57)	62 (96.88)	1 (reference)		1 (reference)	
Sphincter incontinence at 15 days postpartum				0.015		0.003
Yes	3 (4.29)	12 (18.75)	0.19 (0.05–0.72)		0.11 (0.03–0.47)	
No	67 (95.71)	52 (81.25)	1 (reference)		1 (reference)	
Start of sexual relations at 15 days postpartum				0.375		0.256
Yes	3 (4.29)	1 (1.56)	2.82 (0.29–27.83)		4.14 (0.36–47.91)	
No	67 (95.71)	63 (98.44)	1 (reference)		1 (reference)	
Perineal pain at 3 months				0.044		0.023
Yes	4 (5.71)	11 (17.19)	0.29 (0.09–0.97)		0.23 (0.06–0.81)	
No	66 (94.29)	53 (82.81)	1 (reference)		1 (reference)	
Sphincter incontinence at 3 months				0.490		0.139
Yes	12 (17.14)	14 (21.88)	0.74 (0.31–1.74)		0.47 (0.18–1.27)	
No	58 (82.86)	50 (78.13)	1 (reference)		1 (reference)	
Exercise at 3 months				0.197		0.130
Yes	34 (48.57)	24 (37.50)	1.57 (0.79–3.14)		1.75 (0.85–3.62)	
No	36 (51.43)	40 (62.50)	1 (reference)		1 (reference)	
Normalization of sexual intercourse at 3 months				< 0.001		< 0.001
Yes	49 (71.01)	23 (37.10)	4.15 (2.00–8.64)		4.78 (2.14–10.64)	
No	20 (28.99)	39 (62.90)	1 (reference)		1 (reference)	

^a^ aOR: OR adjusted for civil status and attendance during pregnancy at an education course on pregnancy, delivery and newborn care

Table 3 shows the results with the variables measured continuously. The pain manifested by women at 24 h postpartum was scored as 4.4 ± 0.3, compared to 3.4 ± 0.3 by those of the continuous suture group (*p* = 0.011). Three months after the suture was placed pain was assessed in the perineal area, with a score of 0.7 ± 0.2 in the reference group, compared to 0.2 ± 0.2 in the intervention group (*p* = 0.030). There were no significant differences regarding the days after delivery to restart sexual intercourse, although it was higher in the reference group.

**Table 3:** Type of suture and effects on pain score and sexual activity (continuous variables)

Variables	Univariate analysis			Multivariate analysis^a^		
	Continuous suture m (SEM)^b^	Interrupted suture m (sem)	*p* value	Continuous suture m (SEM)	Interrupted suture m (SEM)	*p* value
Perineal pain at 2 h post delivery	1.7 (0.3)	2.3 (0.3)	.142	1.7 (0.3)	2.3 (0.3)	0.208
Perineal pain at 24 h post delivery	3.5 (0.3)	4.4 (0.2)	0.009	3.4 (0.3)	4.4 (0.3)	0.011
Perineal pain at 15 days post delivery	0.8 (0.2)	1.9 (0.3)	0.001	0.8 (0.2)	1.9 (0.2)	0.001
Perineal pain at 3 months	0.2 (0.1)	0.7 (0.2)	0.034	0.2 (0.2)	0.7 (0.2)	0.030
Days till sexual activity ^c^	41.8 (2.4)	48.1 (2.3)	0.060	41.8 (2.4)	48.1 (2.5)	0.072
Days till first intercourse	46.1 (2.2)	50.8 (2.2)	0.137	46.2 (2.2)	50.8 (2.3)	0.168

^a^ Variables adjusted in the multivariate analysis: civil status and attendance during pregnancy to an education course on pregnancy, delivery and newborn care

^b^ m (Mean): SEM (standard error of the mean)

^c^ Sexual activity: refers to caresses, sex games, masturbation and sexual intercourse

## Discussion

Women who had a continuous suture repair showed less pain at 24 h and required less analgesia. The pain was also less at 15 days and at 3 months postpartum. The women in the intervention group also had a lower rate of urinary incontinence at 15 days after delivery. There were no differences in pain at 2 h, neither in the rate of wound dehiscence, nor in the time elapsed for the initiation of sexual relations.

As these were eutocic deliveries, all deliveries were assisted by midwives. However, we do not believe that the type of professional who attends the delivery would influence these results, and therefore if this type of suture had been performed by a gynecologist the results would not have been affected as long as they were eutocic deliveries.

Randomization did not achieve complete balance in all the variables between the two groups. Unbalanced variables were civil status and attendance during pregnancy at an education course on pregnancy, delivery and newborn care. These differences were corrected using multivariate analysis. As marital status and education received during pregnancy may affect the participants’ answers on subjective outcomes, such as pain, the results were adjusted for these two variables too, which did not modify the results of the crude analysis. Another limitation that may exist is that the midwives who collected the data knew the group to which the women belonged, which could affect the collection of pain data and to a lesser extent the identification of incontinence. An attempt was made to reduce this potential source of bias with an objective questionnaire in which the midwives did not influence data collection and using a pain scale—the verbal rating scale (VRS) [15].

A strength of this study was that the women did not know the suture technique used (simple blinding) and therefore their answers could not be influenced by knowing the intervention. In the exclusion criteria, parity was taken into account since it has an influence on the discomfort and problems in the postpartum period [16].

Despite a restricted episiotomy policy, episiotomy rates are high (around 50% in both groups) in line with the results of other authors [1, 3].

A 2008 meta-analysis conducted by Kettle et al. [17] which included seven studies concluded, although not significantly, that the severity of pain up to the tenth day was considerably less with the continuous repair method, and that this difference was maintained up to 12 months after of childbirth, in line with our results. One of the included studies was that of Fleming [18] in 1990, in which they suggested that the difference in pain could be caused by the edema produced from the increased pressure in the sutures. A priori this might explain the lower levels of pain observed in our study group at 24 h and 15 days postpartum. This difference was not detected in the first 2 h, possibly because this edematous reaction had not yet occurred; however, we do not believe that this could be the reason that explains our results with respect to pain at 3 months.

Valenzuela et al. [11] in Spain and Kindberg et al. [19] in Denmark conducted randomized studies of continuous compared to interrupted sutures for perineal repair, both with a sample of 400 women in two groups evaluating the level of pain and the use of analgesia on day two, day ten, and at 3 months postpartum. These studies did not observe any significant difference between the two groups, which to some extent is contrary with our results which showed a lower use of analgesia in the first 24 h. In Turkey, Kokonali et al. [12] conducted a trial in which 160 women were included and concluded that the pain with a continuous suture was significantly less severe than with an interrupted suture repair technique. In this trial, pain was assessed with the performance of different activities. However, it did not take into account parity, and only women who had undergone an episiotomy were included, unlike the study by Valenzuela et al. [11] who evaluated second-degree tears and episiotomies.

The time difference between a continuous suture repair and an interrupted suture repair was 4 min. Valenzuela et al. [11] demonstrated that a continuous suture required 1 min less than interrupted sutures. Results from other authors also report similar findings [ 12, 17]. Another significant difference was the use of less suture material in the continuous repair compared with the repair using interrupted sutures, with a difference of more than one packet. This was also reported in other studies, such as Hasanpoor et al. [8] in Iran, Valenzuela et al. [11] in Spain and Kokonali et al. [12] in Turkey.

Although the commencement of sexual relations was almost 1 week earlier in women with a continuous suture and the mean difference in days till first sexual intercourse was approximately 5 days compared with the continuous suture group, this difference was not significant between groups. Shrivastava et al. [14] and Valenzuela et al. [11] reported the opposite results, with a later re-initiation of sexual relations in the group with continuous sutures. Our results showed that women with a continuous suture had a higher frequency of having normalized their sexual relations at 3 months postpartum.

In our study, we observed less cases of urinary incontinence at both 15 days and at 3 months in women with a continuous suture. We were unable to compare this last result as it has not been analyzed in any previous study.

### Implications for future research

To address significant knowledge gaps in this area, research is needed that: [1] identifies the impact of the type of suture on all types of vaginal delivery, including instrumental deliveries and in multiparous women [2]; identify or, if necessary, develop measures of impact of care in this area [3]; identify the factors that influence professionals to perform one type of suture or another [4]; develop and evaluate the efficacy of continuous suture of the perineal lesion to reduce the negative impact of this and its comorbidities on women in instrumental deliveries [5]; quantify the direct medical costs associated with the comorbidities associated with the type of suture that is performed and identify the factors that influence these costs; and [6] quantify and fully explore the indirect costs of the type of suture. Research that addresses these objectives will provide the empirical basis necessary to improve the quality of life of women.

## Conclusion

The findings in our study demonstrated lower levels of pain, less need for analgesia at 24 h, and a lower incidence of urinary incontinence at 15 days postpartum in the group of women how had a continuous suture. In addition, less time is needed for a continuous suture repair and the amount of suture required is lower for a continuous repair compared with an interrupted suture technique.

## Supplementary information

**Additional file 1.** Annex A. Description of the intervention.

## Data Availability

The datasets used and/or analyzed during the current study are available from the corresponding author on reasonable request. (Juan Miguel Martinez Galiano, email: juanmimartinezg@hotmail.com).

## Abbreviations

aOR: Adjusted Odds ratios
BMI: Body mass Index
CI: Confidence intervals
cm: Centimeters
g: Grams
m: Mean
mm: Millimeters
OR: Odds ratios
SEM: Standard error of the mean
VRS: Verbal rating scale

## References

[1] Melchor JC, Bartha JL, Bellart J, Galindo A, Miño M, Perales A (2008). Episiotomy in Spain: data from 2006. Progresos de Obstetricia y Ginecología.

[2] Viswanathan M, Hartmann K, Palmieri R, Lux L, Swinson T, Lohr KN (2005). The use of episiotomy in obstetrical care: a systematic review. AHRQ Evidence Report Summaries.

[3] Observatorio de Salud de la mujer y del Sistema Nacional de Salud. Dirección General Agencia de Calidad del Sistema Nacional de Salud. Ministerio de Sanidad y Consumo. Strategy for attendance at the Normal Delivery in the National Health System [Estrategia de Atención al Parto Normal en el Sistema Nacional de Salud.] Madrid: Ministerio de Sanidad y Consumo; 2007.

[4] Blondel B, Alexander S, Bjarnadóttir RI, Gissler M, Langhoff-Roos J, Novak-Antolič Ž (2016). Variations in rates of severe perineal tears and episiotomies in 20 European countries: a study based on routine national data in euro-Peristat project. Acta Obstet et Gynecol Scand.

[5] Leeman L, Rogers R, Borders N, Teaf D, Qualls C (2016). The effect of Perineal lacerations on pelvic floor function and anatomy at 6 months postpartum in a prospective cohort of nulliparous women. Birth.

[6] Kudish B, Sokol RJ, Kruger M (2008). Trends in major modifiable risk factors for severe perineal trauma, 1996-2006. Int J Gynaecol Obstet.

[7] Fernando R, Sultan AH, Kettle C, Thakar R, Radley S (2006). Methods of repair for obstetric anal sphincter injury. Cochrane Database of Syst Rev.

[8] Hasanpoor S, Bani S, Shahgole R, Gojazadeh M (2012). The effects of continuous and interrupted episiotomy repair on pain severity and rate of Perineal repair: a controlled randomized clinical trial. J Caring Sci.

[9] Morano S, Mistrangelo E, Pastorino D, Lijoi D, Costantini S, Ragni N (2006). A randomized comparison of suturing techniques for episiotomy and laceration repair after spontaneous vaginal birth. J Minim Invasive Gynecol.

[10] Signorello LB, Harlow BL, Chekos AK, Repke JT (2001). Postpartum sexual functioning and its relationship to perineal trauma: a retrospective cohort study of primiparous women. Am J Obstet Gynecol.

[11] Valenzuela P, Saiz Puente MS, Valero JL, Azorin R, Ortega R, Guijarro R (2009). Continuous versus interrupted sutures for repair of episiotomy or second-degree perineal tears: a randomised controlled trial. BJOG..

[12] Kokanalı D, Ugur M, Kokanalı MK, Karayalcın R, Tonguc E (2011). Continuous versus interrupted episiotomy repair with monofilament or multifilament absorbed suture materials: a randomised controlled trial. Arch Gynecol Obstet.

[13] Kettle C, Dowswell T, Ismail KM (2012). Continuous and interrupted suturing techniques for repair of episiotomy or second-degree tears. Cochrane Database Syst Rev.

[14] Shrivastava D, Sarkar S. Continuous versus interrupted sutures for repair of episiotomy or second-degree perineal tears: A randomised controlled trial. Int J Sci Res. 2018;6(6). 10.1111/j.1471-0528.2008.02056.x 19187377

[15] Pardo P, Muñoz T, Chamorro C (2006). Monitoring pain. Work group recommendations for analgesia and sedation of the SEMICYUC. Med Int.

[16] Martínez-Galiano JM, Hernández-Martínez A, Rodríguez-Almagro J, Delgado-Rodríguez M, Gómez-Salgado J (2019). Relationship between parity and the problems that appear in the postpartum period. Sci Rep.

[17] Kettle C, Hills RK, Ismail KMK. Continuous and individual interrupted sutures for repair of episiotomy or second-degree tears. Cochrane Libr. 2008;4 Available at: http://www.update-software.com. 10.1002/14651858.CD000947.pub2 17943747

[18] Fleming N (1990). Can the suturing method make a difference in postpartum, perineal pain. J Nurse Midwifery.

[19] Kindberg S, Stehouwer M, Hvidman L, Henriksen TB (2008). Postpartum perineal repair performed by midwives: a randomised trial comparing two suture techniques leaving the skin unsutured. BJOG.

