# Supplementary material for: Continuous versus discontinuous suture in perineal injuries produced during delivery in primiparous women: a randomized controlled trial
Source: BMC Pregnancy Childbirth. 2019 Dec 16;19:499. doi: 10.1186/s12884-019-2655-2 (PMC6916034; doi:10.1186/s12884-019-2655-2)
Supplement: Supplementary file 1 — Additional file 1. Annex A. Description of the intervention. [file 12884_2019_2655_MOESM1_ESM.docx]

**ANNEX A**. Description of the intervention

**Group A. *Continuous suture technique*:**

Suture material: “Safil quick” 2/0, polyglycolic acid, braided, coated, rapid absorption. All parts of the perineal lesion will be sutured with the same suture thread.

* Vaginal mucosa suture: The first stitch is placed 0.5-10 millimeters (mm) above the apex and secured with a double knot to ensure hemostasis. A continuous unlocked suture was placed approximately half a centimeter from the edges of the wound and was run from the apex to the hymenal ring making sure to include the vaginal mucosa and rectovaginal fascia.

* The perineal muscles were approximated using simple continuous unlocked sutures ensuring to obliterate the dead space and leaving 0.5 centimeters (cm) margin for skin suturing.

* Skin suture: the skin should be correctly approximated, but without tension, as a skin suture can increase the incidence of perineal pain in the first postpartum months. Continue from the superficial muscle until the skin is approximated and finish with a subcutaneous/intradermal stitch.

**Group B. *Interrupted suture technique*:**

* Vaginal mucosa suture: suture material: “Safil quick” 0/0, polyglycol acid, braided, coated, rapid absorption. Identify the wound apex. The anchor point of the suture should be 1cm above the apex. Place a continuous suture from the angle until the hymenal ring. It should include the vaginal mucosa and the rectovaginal fascia. A continuous suture is placed with locking stitches till the hymenal ring. Tie off with knots, to the right and to the left and finish with another to the right. Cut the thread at 1cm.

* Perineal muscle suture: identify the perineal muscles on both sides of the lesion and approximate them with separate interrupted sutures of “Safil quick” 0/0.

* Skin suture: The end result should have skin approximated but without tension, as a skin suture can increase the incidence of perineal pain in the first postpartum months. Interrupted sutures of “Safil quick” 2/0 are placed, avoiding tension.
